# Low-angle Paramedian Approach versus Midline Approach for Remote Access Epidural Blood Patch: A Case Report

**DOI:** 10.4274/TJAR.2026.262615

**Published:** 2026-10-30

**Authors:** Argun Pire, Can Eyigör, Burcu Özalp, Meltem Uyar

**Affiliations:** 1 Ege University Faculty of Medicine Department of Anaesthesiology and Reanimation, İzmir, Türkiye

**Keywords:** Algology, epidural blood patch, intracranial hypotension, pain, regional anaesthesia

## Abstract

While mid-thoracic epidural blood patch leaks (T7-T8) require targeted epidural blood patches, direct thoracic puncture carries a significant risk of spinal cord injury due to the narrow epidural space. To mitigate this risk, a safety-oriented remote-access strategy from the T12-L1 level, with cranial advancement of the catheter, was employed to prevent direct thoracic spinal cord injury. We present a 46-year-old male with a T7-T8 leak in whom a standard midline approach failed due to poor catheter steerability. By adopting a low-angle (<30°) paramedian approach, we achieved precise ipsilateral catheter positioning; this technical modification provides optimized vector alignment and a tangential epidural trajectory for unilateral thoracic leaks. This technical modification may improve maneuverability and safety for unilateral thoracic leaks, compared to standard midline techniques, and appears to facilitate an optimized tangential trajectory in this case.

**Main Points**

• Mid-thoracic cerebrospinal fluid leaks pose high procedural risk due to a narrow epidural space.

• Midline approaches often limit catheter steerability during remote access.

• A low-angle (<30°) paramedian approach aligns with the epidural axis, enabling easier cephalad advancement.

• This technique is an effective alternative for precise targeting in difficult interventions.

## Introduction

Spontaneous intracranial hypotension is characterized by reduced cerebrospinal fluid (CSF) volume and can mimic Arnold-Chiari malformation (ACM) through “brain sagging”. In post-ACM-surgery patients, low CSF pressure causes downward brainstem displacement, leading to diagnostic confusion and potentially unnecessary re-operation.^1^ While the epidural blood patch (EBP) is the gold standard,^2^ direct mid-thoracic (T5-T9) needle entry risks spinal cord injury due to narrow epidural anatomy.^3^ To mitigate this, a safety-oriented remote-access strategy using the T12-L1 interspace, with cranial advancement of the catheter to the T7-T8 target, is preferred. However, unilateral targeting remains challenging via standard midline approaches. This report documents technical success using a low-angle paramedian approach after failure of the midline technique.

## Case Report

Written informed consent was obtained. A 46-year-old male who underwent posterior fossa decompression for ACM Type-1 in 2021 presented three years postoperatively with progressive orthostatic headache, balance disturbance, and a 40-kg weight loss over 18 months. Physical examination showed hoarseness, horizontal diplopia, mild left-sided clumsiness or latent paresis, wide-based ataxic gait, and a positive left Babinski reflex.

Cranial magnetic resonance imaging demonstrated findings highly suggestive of intracranial hypotension: tonsillar descent to C2, diffuse dural contrast enhancement, narrowed prepontine cistern, and prominent pituitary gland. Dynamic computed tomography myelography identified an active CSF leak at the right T7-T8 neural foramen (Figure 1). Following failure of conservative management, an EBP was planned.

### Interventional Procedure

Performed prone under conscious sedation (2 mg midazolam, 50 µg fentanyl) and intermittent fluoroscopy. The T12-L1 interspace was selected for remote entry to avoid thoracic cord injury.

### Standard Midline Approach

The epidural space was accessed via a midline interlaminar approach using an 18-gauge, 9-cm Tuohy needle (B. Braun, Melsungen, Germany), confirmed by loss of resistance to saline. A standard 20-gauge polyamide catheter consistently deviated into the left lateral (contralateral) space on anteroposterior (AP) and lateral fluoroscopy and failed to advance past T10. The injection of 2 mL of non-ionic contrast medium (iohexol, 300 mg I/mL) confirmed contralateral spread (Figure 2). A right-sided low-angle paramedian approach was subsequently performed at the same level. The Tuohy needle was inserted at the right paramedian line, forming an angle of less than 30 degrees relative to the skin. This shallow trajectory produced a tangential epidural path parallel to the longitudinal axis of the epidural space. Due to this optimized vector alignment, the catheter was advanced cranially along the ipsilateral dorsal gutter to the target at the T8 level without resistance. Catheter position and strictly unilateral contrast spread were confirmed using AP and lateral fluoroscopic projections (Figure 3). The geometric and vector differences between these two needle trajectories are schematically illustrated in Figure 4.

Following fluoroscopic confirmation and contrast spread, 10 mL of autologous venous blood was injected. Following the injection, the catheter was removed, and the patient was kept on strict supine bed rest for 4 hours and underwent regular neurological assessments. The patient experienced immediate and complete resolution of the orthostatic headache and associated neurological deficits. At the 1-month post-procedural follow-up, the patient remained asymptomatic, with complete restoration to baseline function and no clinical signs of recurrence.

## Discussion

The mid-thoracic region is a high-risk zone for interventional procedures due to the narrowness of the epidural space and the anatomical position of the spinal cord.^4^ In our case, distinguishing remote-access entry level (T12-L1) from the target thoracic leak level (T7-T8) constitutes a safety-oriented strategy explicitly designed to obviate the need for direct mid-thoracic puncture and thereby reduce the risk of direct injury to the thoracic spinal cord. The most critical technical finding presented in this case is the impact of the insertion angle on the steerability of the epidural catheter. In standard midline approaches, entering the epidural space at a steep vertical angle alters the vector alignment, making the cephalad advancement of the catheter unpredictable. This steep trajectory may lead to reduced torque control and increase the potential for interactions with epidural fat or connective tissue structures, which can deflect the catheter tip. Furthermore, the specific orientation of the Tuohy needle bevel during a steep entry profile may steer the catheter into an unfavorable pathway from the outset. A clinical study by Ryu et al.^5^demonstrated that approaching the thoracic epidural space at a more horizontal angle significantly increases catheter threading length without coiling, directly illustrating how steeper tracts impede linear advancement. In contrast, the paramedian, shallow-angle entry (<30°) used in spinal cord stimulation implantation ensures parallel alignment with the longitudinal epidural axis, thereby creating a tangential trajectory within the epidural space.^6^ This configuration provides an optimized vector alignment, allowing unimpeded insertion of the epidural catheter into the caudal end of the epidural segment, effectively preventing it from impacting the bony lamina above. As a result, this technique increases the likelihood of the catheter remaining on its intended trajectory and significantly reduces the risk of unintended lateral or anterior deviation, venous puncture, and paraesthesia.^7^ Furthermore, achieving complete clinical recovery with only 10 mL of autologous blood is a noteworthy aspect of this case. Unlike conventional patches where the injectate spreads diffusely over multiple segments, the blood in this case was deposited locally via the catheter tip and remained directly at the T7-T8 level. Conventional lumbar or non-targeted epidural blood patches frequently require larger injectate volumes, often around 20 mL, to ensure sufficient cranial migration and volume distribution to reach distant mid-thoracic leaks.^2^ Our experience suggests that accurate ipsilateral targeting within the dorsal gutter may allow effective treatment using lower blood volumes in selected patients, potentially minimizing the risks of transient post-procedural back pain or excessive intrathecal pressure spikes associated with large-volume injections. Nonetheless, given the single-case nature of this report, these observations must be interpreted with caution. Definitive comparative conclusions regarding the technical superiority or safety profile of this approach cannot be firmly established, and prospective comparative studies are required to validate these potential benefits.

## Conclusion

Catheter-guided EBP at T12-L1 appears to be a viable approach to reduce complications from mid-thoracic CSF leaks. While midline steering is often limited for unilateral targets, a low-angle paramedian approach appeared to facilitate safe catheter advancement and improve maneuverability, enabling effective access to the targeted side.

## Ethics

**Informed Consent:** Written informed consent was obtained from the patient for the publication of this case report.

## Figures and Tables

**Figure 1:**
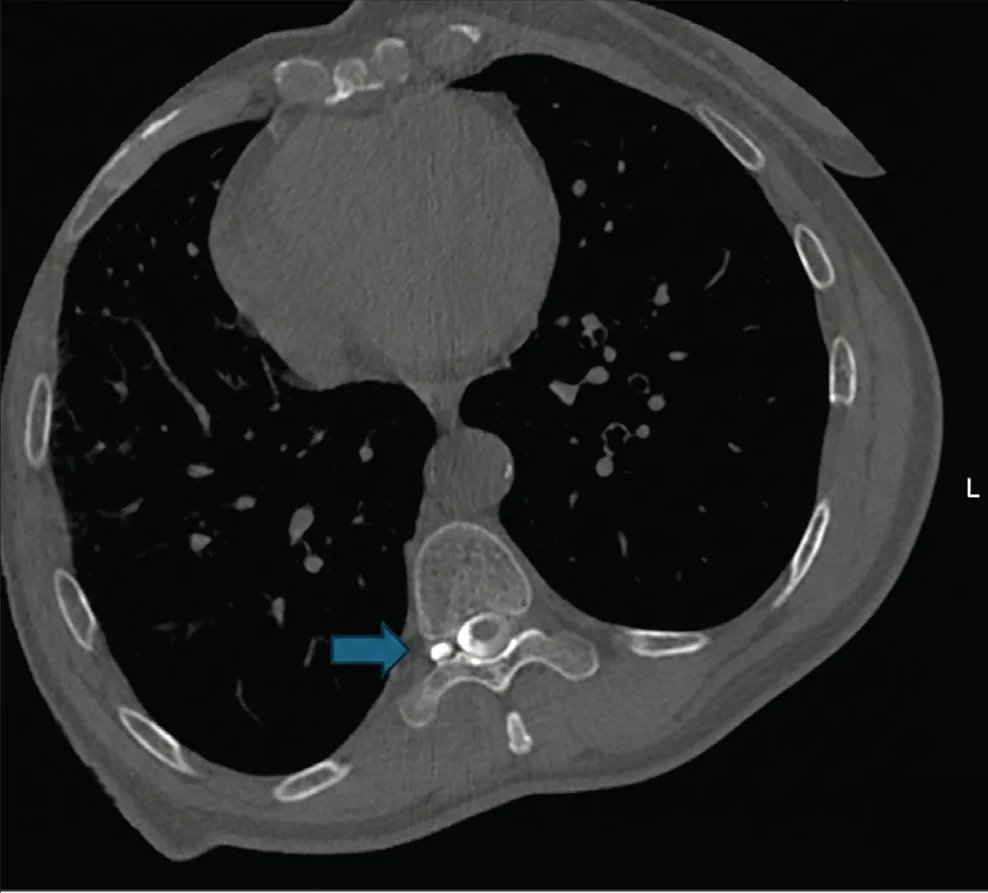
Axial dynamic computed tomography (CT) section showing a cerebrospinal fluid (CSF) leak on the right side of the spinal canal (area indicated by the arrow).

**Figure 2:**
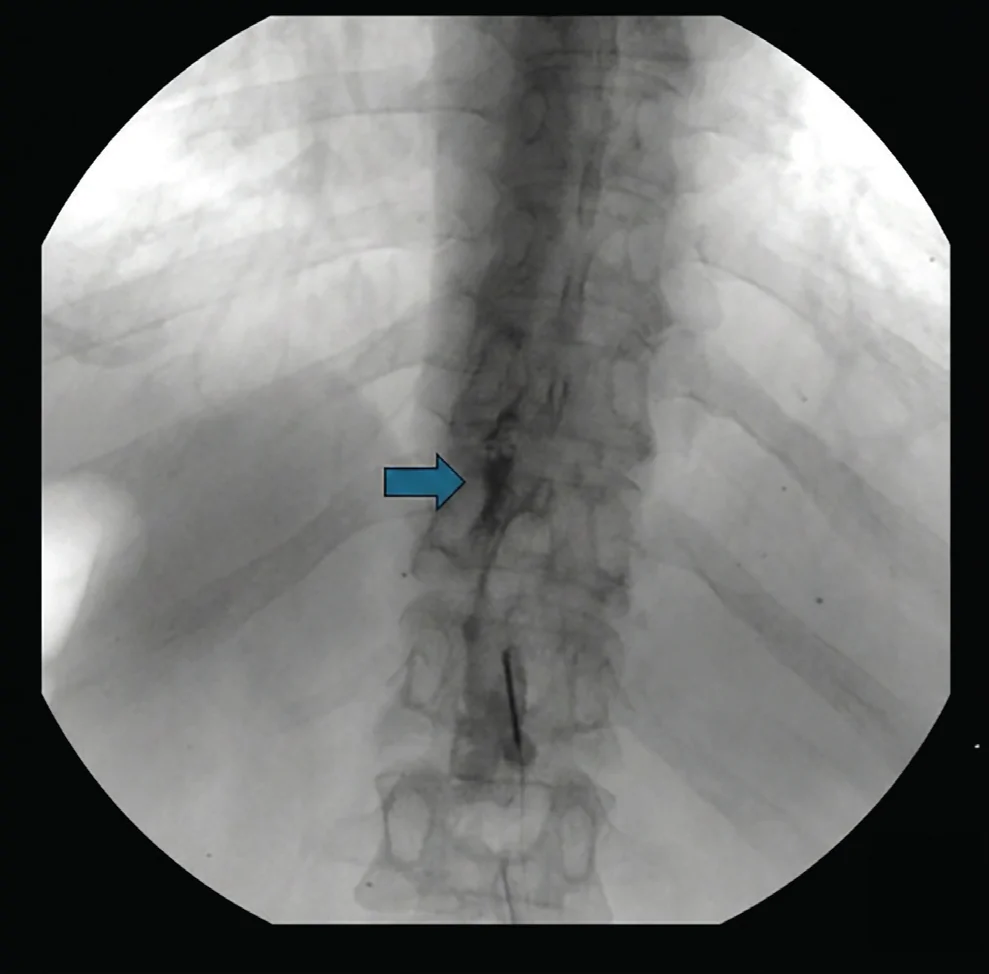
Fluoroscopic view of the standard midline approach. Upon entering the epidural space via the T12-L1 midline, the catheter consistently deviates toward the left side, preventing any corrective steering toward the right-sided target. Epidurography clearly demonstrates a contralateral contrast spread pattern, visually defining the technical failure to achieve targeted unilateral lateralization due to the steep insertion geometry.

**Figure 3:**
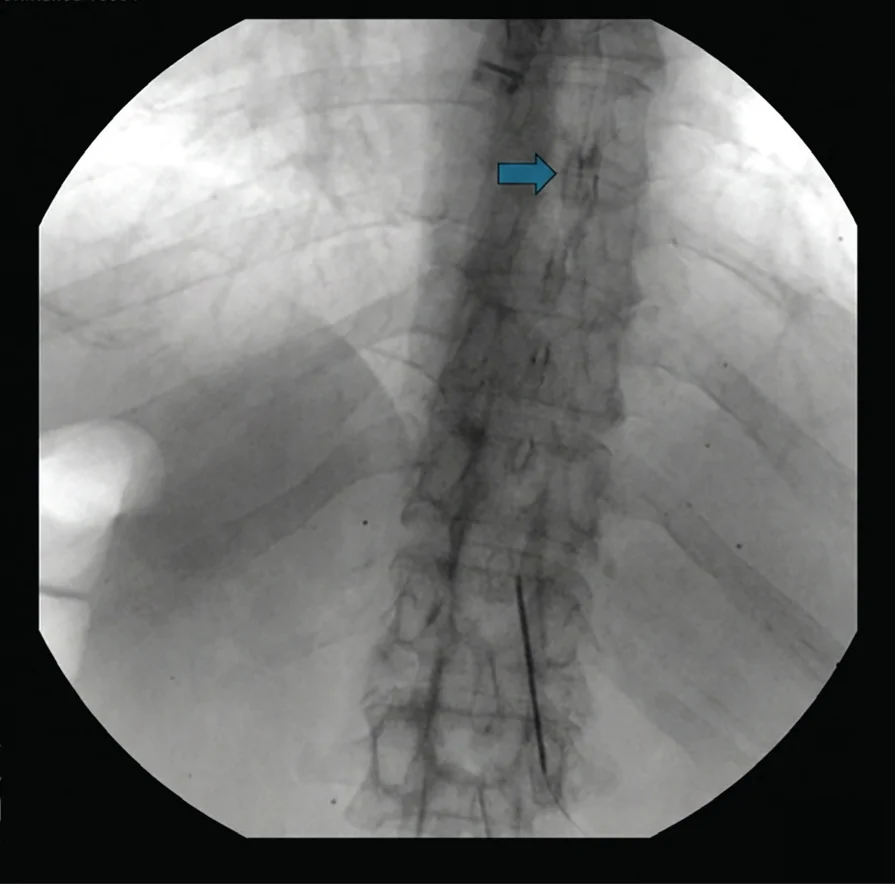
Fluoroscopic view of the low-angle paramedian approach. Utilizing a right-sided oblique entry angle, the catheter advances smoothly through the right epidural space into the dorsal gutter, with the tip precisely positioned at the T8 level. Epidurography demonstrates a strictly ipsilateral contrast spread pattern, confirming highly accurate and successful anatomical targeting of the right-sided thoracic leak.

**Figure 4:**
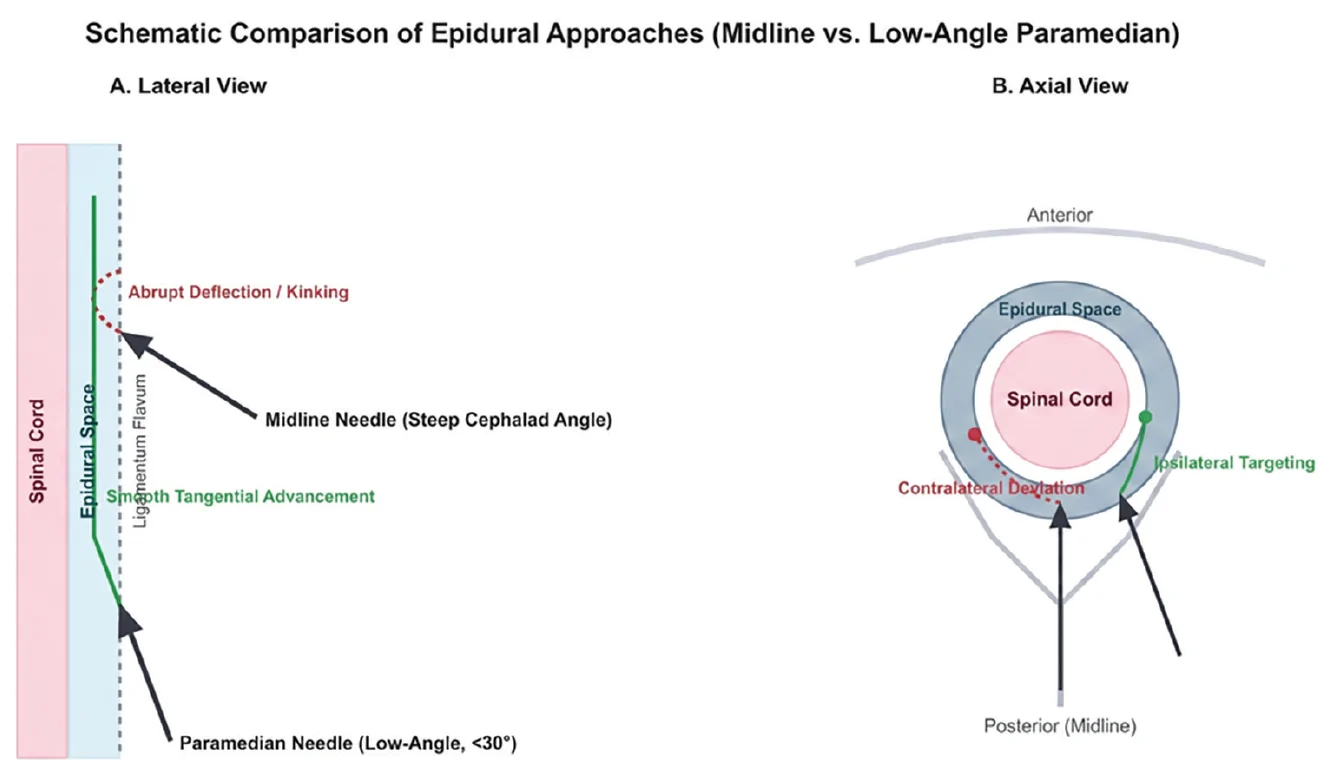
Schematic illustration highlighting the direct relationship between needle tract geometry, catheter behavior, and the resulting contrast distribution patterns. (A) Lateral View: The steep midline approach forces an abrupt deflection of the catheter upon entry, leading to kinking and unpredictable navigation. Conversely, the low-angle (<30°) paramedian approach establishes a tangential trajectory parallel to the longitudinal epidural axis, facilitating smooth, unobstructed cranial advancement. (B) Axial View: The midline approach results in an uncontrolled vector that drives the catheter into the opposite side, producing a contralateral contrast spread pattern. In contrast, the right-sided paramedian approach optimizes the directional vector directly into the right dorsal gutter, achieving the definitive ipsilateral contrast spread pattern that constitutes the central technical success of this technique.
